# Correlation of In-situ Hand Anatomy With Point of Care Ultrasound

**DOI:** 10.7759/cureus.40228

**Published:** 2023-06-10

**Authors:** Mario Loomis, Gilberto Kistner, Daniel Prabahkar, Jonathon H Hines, Teresa Loomis, Jaime Hinojosa

**Affiliations:** 1 Department of Clinical Anatomy, Sam Houston State University College of Osteopathic Medicine, Conroe, USA

**Keywords:** hand surgery, cadaver hand, in-situ anatomy, hand anatomy, point of care ultrasound

## Abstract

The effective use of point-of-care ultrasound for the diagnosis and treatment of hand conditions is dependent upon a thorough understanding of its anatomic bases. To facilitate this understanding, in-situ cadaveric hand dissections were correlated with handheld ultrasound images in the palm focusing on key areas of clinical relevance. The palms of an embalmed cadaver were dissected, minimizing the reflection of structures whenever possible to emphasize normal relationships and tissue planes. Point-of-care ultrasound images were obtained from a living hand and correlated with related anatomy in the cadaver. Juxtaposing cadaver structures, spaces, and relationships with the associated ultrasound images, surface hand orientation, and ultrasound probe positioning, a series of images were developed as a guide to correlating in-situ anatomy with point-of-care ultrasound in the hand.

## Introduction

Point-of-care ultrasound in the palmar hand has many applications for common surgical hand conditions [1]. It can assist with minimal incision carpal tunnel surgery [2], the diagnosis of an incomplete carpal tunnel release [3], and the diagnosis of injury to the recurrent motor branch of the median nerve [4]. It can help diagnose and treat deep space infections in the hand [5,6] and help retrieve proximal ends of flexor tendon lacerations [7,8]. It can guide percutaneous treatments of stenosing tenosynovitis [9,10], foreign body removal, and the recognition of anatomical variations. The correlation of ultrasound images with gross anatomy can help hand surgeons, therapists, or primary care and emergency physicians training to incorporate point-of-care ultrasound into their patient care. Likewise, it can help medical students, whose time spent in gross anatomy has been steadily reduced in recent years [11], to better understand the complex structural relationships in the palm. Supplemental correlations of anatomy with surgical and clinical scenarios have been shown to improve student understanding [12]. In this project, multiple ultrasound images were correlated with palmar anatomy dissections, focusing on common sites of clinical relevance, as a supplement to help students learning hand anatomy and ultrasound.

## Technical report

Detailed dissections of the palms were performed on an embalmed cadaver with the addition of tumescence to help maintain normal anatomical relationships. Tumescence is simply the intermittent infusion of a wetting solution into the interstitial space to make it easier to separate and spare delicate structures. The solution was introduced through a blunt-tipped cannula and consisted of 80% water, 18% propylene glycol, 0.4% phenoxyethanol, and 1.6% Sanisol 7® detergent disinfectant (Trinity Fluids, Harsens Island, MI). The transverse carpal ligament was kept intact on one side, and other structures kept in-situ whenever possible to demonstrate their usual relationships with adjacent structures and spaces. For example, the palmar skin was reflected in one piece, no tendons were transected, and the boundaries of the midpalmar and thenar spaces were maintained. The correlations focused on clinically relevant sites such as those involved with infection, tendon injuries, stenosing tenosynovitis, arthritis, nerve compression, and foreign bodies.

**Figure 1:**
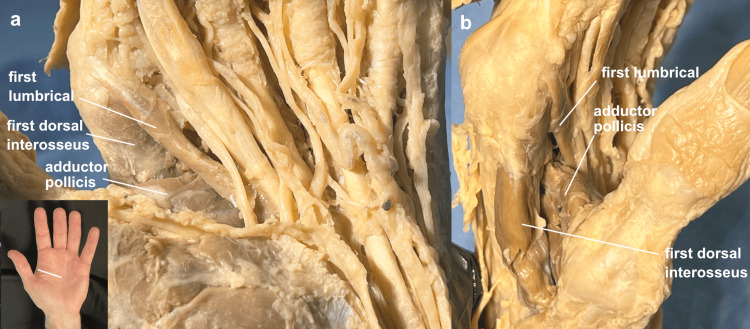
First webspace muscles First webspace muscles in a left hand: (a) palmar view, (b) radial view

In the cadaver, the muscle fibers of the adductor pollicis and the first dorsal interosseus muscles were demonstrated to run at right angles to each other, those of the first lumbrical running slightly obliquely to those of the adductor pollicis (Figure 1).

**Figure 2:**
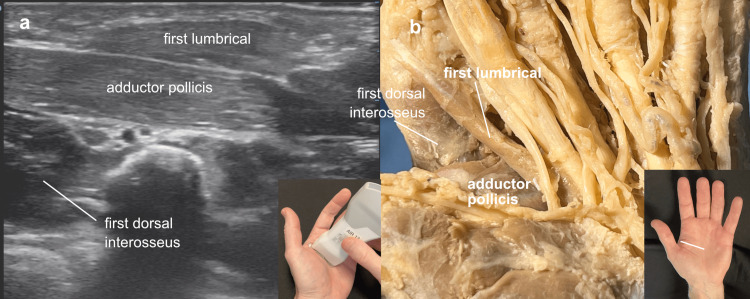
Adductor orientation Adductor orientation: (a) Ultrasound view over the palmar first webspace of a left hand, the probe marker directed toward the thumb (left) for easier correlation with the cadaver view, demonstrating long axis views of the adductor pollicis and first lumbrical muscles and a short axis view of the first dorsal interosseus muscle, (b) cadaver demonstration of the same structural relationships.

These relationships were appreciated using handheld ultrasound imaging in a left palm, aligning the probe along the fibers of the adductor pollicis muscle to provide a long axis view of the muscle. While the convention is to orient the probe marker to the patient’s right, for our purpose, we oriented it towards the patient’s left (the thumb) so that the left side of the ultrasound image would correspond to the left side of the cadaver image. (The probe marker side is displayed on the left side of the ultrasound screen.) The fibers of the adductor pollicis in the left hand were readily apparent on ultrasound, though it was usually necessary to increase the gain to see the fibers clearly. These longitudinal fibers seen in the long axis view of the adductor pollicis were contrasted with a cross-section of the first dorsal interosseus fibers seen in the short axis view. The first lumbrical was similar in appearance to the adductor, running slightly obliquely to it and more superficial, noted in both the ultrasound and cadaver image (Figure 2).

**Figure 3:**
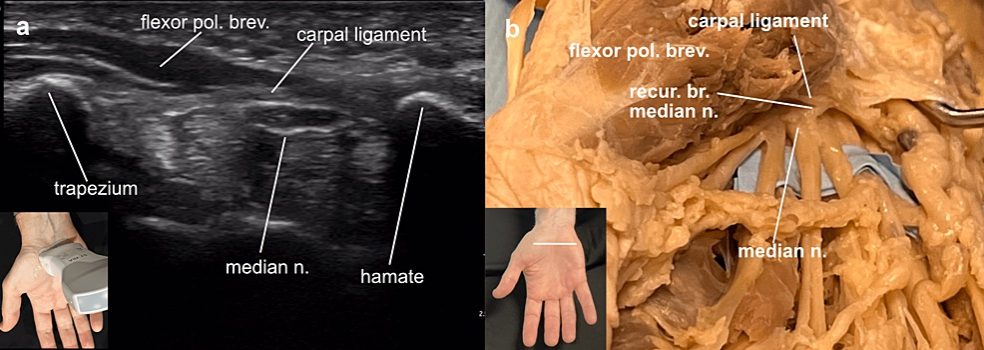
Transverse carpal ligament Transverse carpal ligament: (a) ultrasound view of the right hand with the probe marker directed toward the patient’s right (the thumb) revealed the transverse carpal ligament between the trapezium and hamate bones, (b) cadaver view looking into the carpal tunnel from the palm, demonstrating the transverse carpal ligament, the median nerve with its recurrent motor branch, and the flexor pollicis brevis muscle overlying the ligament.

Sliding the probe along the adductor medially into the palm demonstrated the flexor tendons to the index, long, ring, and little fingers. The tendons were then followed proximally to the opening of the carpal tunnel. The tubercle of the trapezium and the hook of the hamate were demonstrated as bright hyperechoic landmarks with the transverse carpal ligament, the roof of the carpal tunnel, running between them. In the cadaver view, the location of the transverse carpal ligament was noted in the palm with the flexor pollicis brevis muscle overlying it. In the ultrasound image, this muscle was seen as a hypoechoic area directly superficial to the radial side of the transverse carpal ligament. Deep to that ligament, the hyperechoic rim around the median nerve was demonstrated (Figure 3).

**Figure 4:**
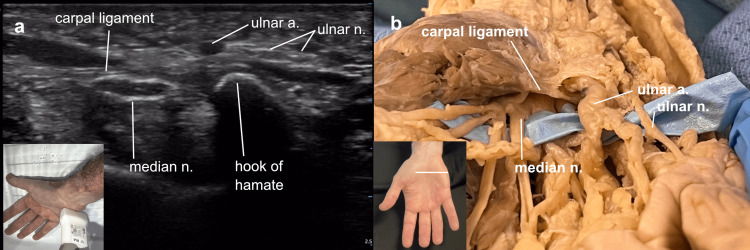
Ulnar (Guyon’s) canal Ulnar (Guyon’s) canal: (a) The ultrasound view of a right hand obtained in a water bath to avoid air artifact demonstrates the ulnar canal superficial to the transverse carpal ligament, (b) the cadaver view demonstrating the degree to which the ulnar canal overlies the carpal tunnel.

By scanning proximally and distally over the carpal tunnel, the flexor pollicis longus tendon was seen diverging radially toward the thumb at the distal opening of the tunnel. In areas of the hand where it was difficult to maintain contact with the entire length of the probe, the ultrasound investigation was carried out with the hand submerged in a water bath. This allowed for scanning over narrower areas without air artifact. In addition, it avoided the compression of the superficial structures often needed to obtain good contact with the probe. With this arrangement, the area of the ulnar (Guyon’s) canal was explored. The roof of the canal was seen to be comprised of the palmar fascia, flexor retinaculum, and palmaris brevis muscle overlying the ulnar artery and nerve, distinct from and superficial to the roof of the carpal tunnel, demonstrated to be the transverse carpal ligament. A scan directly over the ulnar canal demonstrated these relationships (Figure 4).

**Figure 5:**
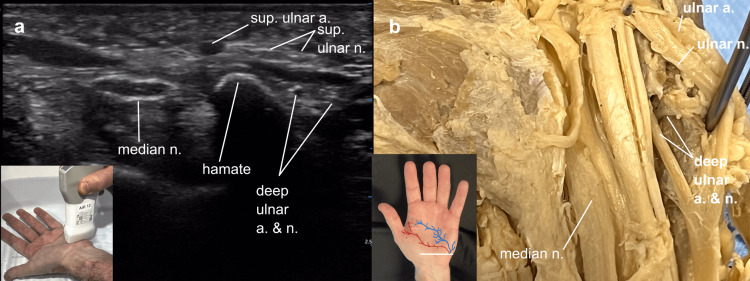
Deep ulnar branches Deep ulnar branches: (a) Ultrasound view of a left hand over the ulnar (Guyon’s) canal with the probe marker directed towards the left (thumb) for better correlation with the cadaver view, demonstrating the deep ulnar artery and nerve traveling beneath the hypoechoic hypothenar muscles, (b) cadaver demonstration of the deep ulnar artery and nerve piercing the proximal hypothenar muscles, hand inset image: blue line representing deep ulnar nerve innervating interossei, adductor, and hypothenar muscles, red line representing deep ulnar artery communicating with deep palmar arterial arch.

This same ultrasound view was then correlated with a cadaver illustration of the deep ulnar artery and nerve as they dove into the hypothenar muscles, a clinically significant site as the deep ulnar nerve can be constricted at this location, in addition to the more superficial tunnel. The approximate routes of the deep branches going on to join the deep palmar arterial arch and to innervate hypothenar and multiple intrinsic muscles were illustrated on an external hand image. The hypoechoic area seen in the ultrasound image between the superficial and deep ulnar arteries and nerves, correlated with the cadaver’s hypothenar muscles into which the deep ulnar artery and nerve were seen to enter (Figure 5).

**Figure 6:**
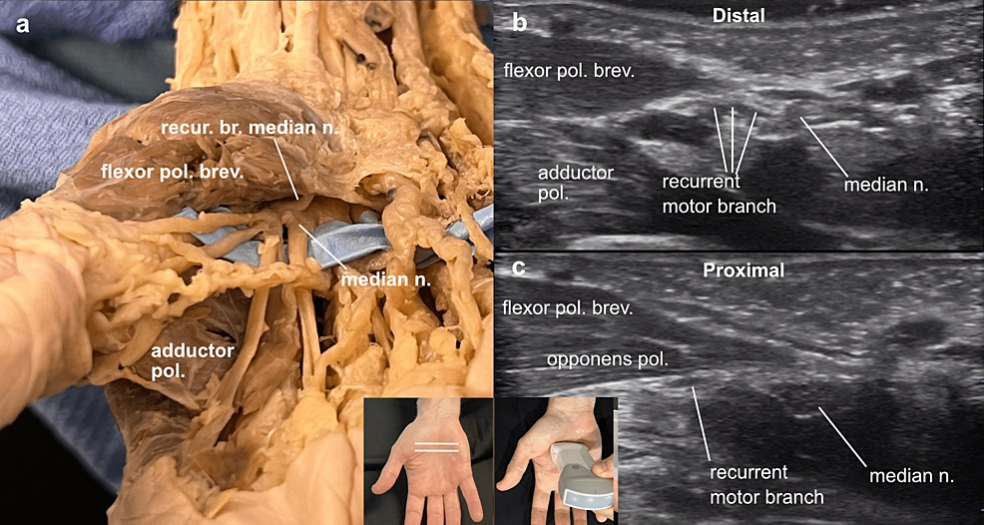
Recurrent motor branch of the median nerve Recurrent motor branch of the median nerve: (a) In the cadaver’s right hand, the recurrent branch was demonstrated looping back from the distal end of the carpal tunnel to innervate the thenar muscles, (b) on ultrasound of a right hand, the probe marker oriented to the right (the thumb), the recurrent branch of the median nerve was followed distal to proximal.

Returning to the carpal tunnel and slowly sliding proximally from the distal opening, the recurrent motor branch of the median nerve was identified and tracked proximally (Figure 6).

**Figure 7:**
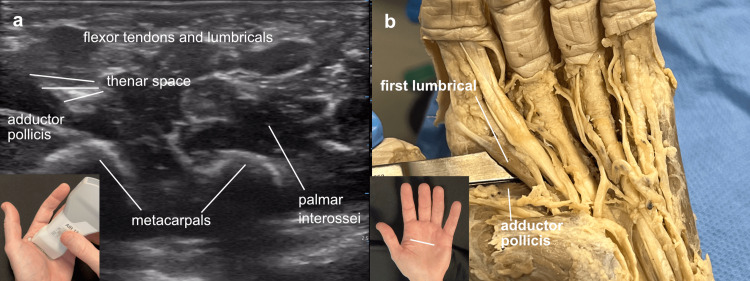
Thenar space Thenar space: (a) Ultrasound view of a left hand with the probe marker oriented to the left for easier correlation with the cadaver view, demonstrates the thenar space superficial to the adductor pollicis and deep to the first lumbrical, (b) the space is entered in the cadaver with blunt dissection between the adductor pollicis and the first lumbrical.

From the carpal tunnel, the probe was slid distally into the palm. The thenar space was located on ultrasound by identifying the area just superficial to the adductor muscle. In the cadaver, after gently advancing a blunt cannula between the adductor and the first lumbrical, additional tumescence fluid was infused and the thenar space demonstrated (Figure 7).

**Figure 8:**
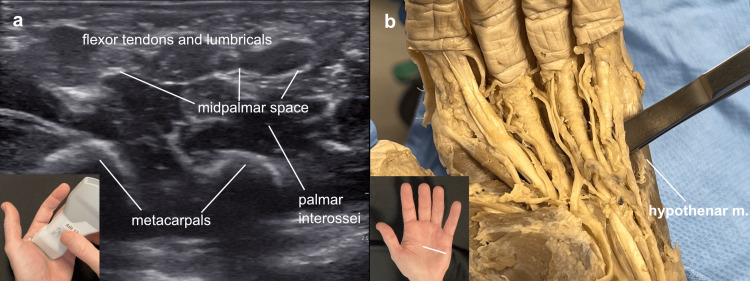
Midpalmar space Midpalmar space: (a) Ultrasound view of a left hand with the probe marker oriented to the left for ease of cadaver correlation, demonstrating the midpalmar space superficial to the interossei, (b) midpalmar space in the cadaver was entered with blunt dissection between the hypothenar muscles and the flexor tendons.

The midpalmar space was approached from the ulnar side of the hand remaining just superficial to the hypothenar muscles and deep to the flexors and lumbricals. Once again, tumescence was infused in the area between the hypothenar muscles and the flexor tendons which helped to open the space in the cadaver, noted to be superficial to the interossei and deep to the flexor tendons. With ultrasound investigation, this same relationship was identified. Having the patient flex their fingers can help delineate the flexor tendons from the interossei (Figure 8).

**Figure 9:**
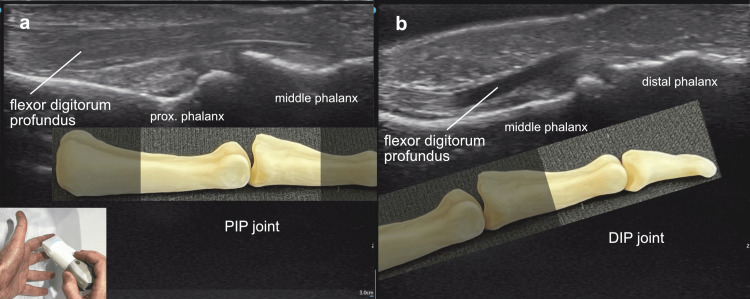
Flexor digitorum profundus Flexor digitorum profundus: (a) Ultrasound scan over the proximal and (b) distal interphalangeal joints of a left index finger in a water bath, the probe marker oriented toward the base of the finger, demonstrated both the flexor tendon and the site of its insertion into the distal phalanx.

With the hand in a water bath, an ultrasound scan along the long axis of the index finger, the probe marker oriented toward the base of the finger, demonstrated the flexor digitorum profundus tendon along its length, over the proximal and distal interphalangeal joints (Figure 9).

**Figure 10:**
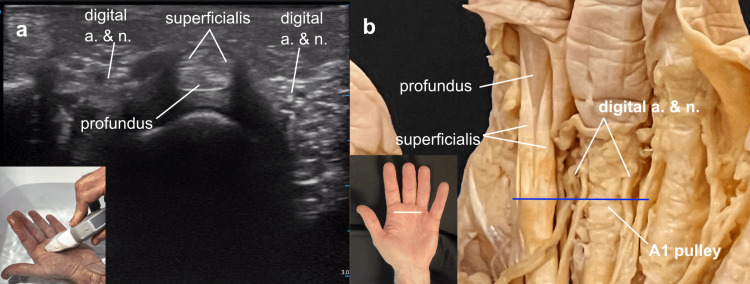
Stenosing tenosynovitis (trigger finger) anatomy Stenosing tenosynovitis (trigger finger) anatomy: (a) Short axis view of the region of the A1 pulley on ultrasound, and (b) in the cadaver demonstrating the relationship between the flexor tendon pulleys and adjacent neurovascular structures.

Turning the probe for a short axis view, still in the water bath, the flexor tendons were demonstrated at the level of the A1 pulleys in the palm, over the metacarpal heads. The flexor digitorum profundus tendon was demonstrated to be deep to the flexor digitorum superficialis tendon at this site, but to pass through the chiasm of the superficialis tendon just a centimeter distal along the digital ray. The close association of the neurovascular bundles to the flexor tendons was seen in both the cadaver and the short axis ultrasound view (Figure 10).

**Figure 11:**
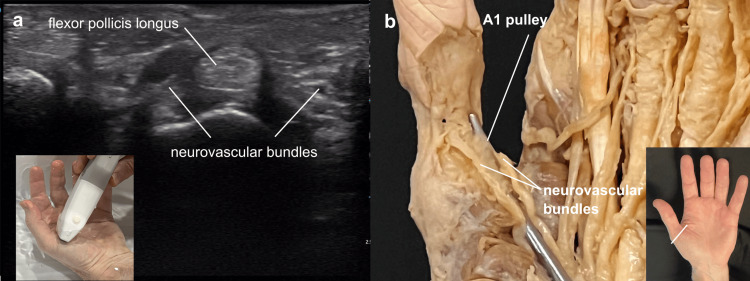
Stenosing tenosynovitis (trigger) thumb anatomy Stenosing tenosynovitis (trigger) thumb anatomy: (a) Short axis ultrasound view of the flexor pollicis longus of the left hand at the level of the A1 pulley, the probe marker oriented to the left to correlate with cadaver image, (b) cadaver view with an instrument passed through the A1 pulley of the thumb.

The probe was placed over the first metacarpophalangeal joint for a short axis view of the A1 pulley of the thumb. The water bath again facilitated scanning at this site. The closeness of the neurovascular bundles to the A1 pulley was evident on both ultrasound and cadaver views (Figure 11).

## Discussion

Recognition of pathology requires familiarity with normal anatomy. Our goal in juxtaposing ultrasound images with gross anatomy images was to make the recognition of normal anatomy more intuitive. Just as clinical correlations have improved student understanding of anatomy [12], ultrasound correlations with in-situ gross anatomy should help with both anatomical and ultrasound understanding. Becoming familiar with a handheld ultrasound exploration of the carpal tunnel and ulnar (Guyon’s) canal could develop or refresh an understanding of the relationships between those two areas, both for precision with surgical or percutaneous interventions, and for evaluation and treatment of traumatic injuries. Identifying the location of the thenar and midpalmar spaces on ultrasound could help in the recognition of abnormal fluid accumulations in those areas. Being able to scan a finger with a healed laceration could pick up a missed partial tendon laceration needing appropriate care and protected motion to heal properly. Familiarity with the short axis ultrasound view of the A1 pulley could help localize the adjacent neurovascular bundles for better precision and safety of a steroid injection to treat stenosing tenosynovitis. For students, self-exploration of the palm with point-of-care ultrasound could help focus the learning of anatomy in terms of interrelationships and clinical applications. While previous work has been done with high-resolution ultrasound images and transversely cut cadaver hands to produce comparable images and identify landmarks [13], our correlation of point-of-care ultrasound with hand structures whose inter-relationships and tissue planes were kept intact, provides an additional perspective from which to learn the complex anatomy of the palm. Limitations of our project include the use of only one cadaver dissection and the scanning of a single patient’s hands. Potential future studies could investigate the correlations with a larger variety of hands scanned and hands dissected. Whether or not and to what degree the correlations actually improve learning of anatomy and point of care ultrasound could be quantified in medical school and residency coursework, and the correlations could also be expanded to include actual clinical cases.

## Conclusions

Multiple point-of-care ultrasound images were correlated with palmar anatomy dissections, focusing on common sites of clinical relevance, as an educational supplement for medical students learning hand anatomy. The juxtaposed images of structures, spaces, and relationships could be referenced as well by those learning point-of-care ultrasound. For all those interested in the palm of the hand, from anatomy students to residents, to allied health practitioners, we offer these images as a supplemental aid to becoming more proficient in the anatomy and point-of-care ultrasound of the palmar hand.
